# Natural Killer (NK) Cell Alloreactivity in Haploidentical Stem Cell Transplantation

**DOI:** 10.3390/cells14141091

**Published:** 2025-07-16

**Authors:** Mar Luis-Hidalgo, José Luis Piñana, Carlos Solano, Dolores Planelles

**Affiliations:** 1 Centro de Transfusión de la Comunidad Valenciana, 46014 Valencia, Spain; planelles_dol@gva.es; 2 Hospital Clínico Universitario de Valencia, 46010 Valencia, Spain; pinana_jos@gva.es (J.L.P.); solano_car@gva.es (C.S.)

**Keywords:** alloreactivity, natural killer, haploidentical hematopoietic stem cell transplantation

## Abstract

This paper conducts a literature review on the role of natural killer cells in haploidentical hematopoietic stem cell transplantation. Theoretical concepts related to *KIR* genes are introduced regarding their structure, nomenclature, genetic organization, polymorphism, and inheritance pattern, types of KIR proteins and receptors, HLA ligands for KIR receptors, and the definition of different NK alloreactivity prediction models for the donor of haploidentical hematopoietic stem cell transplantation and the recipient. These models include the following and consider incompatibility: ligand–ligand, receptor–ligand, gene–gene, and KIR haplotype models or the *KIR-B* donor group. These models consider the presence or absence of specific ligands or receptors and/or *KIR* genes in the donor and recipient to predict alloreactivity. Determining the best model for predicting KIR alloreactivity and its significance in donor selection algorithms for haploidentical transplantation is still under investigation.

## 1. Main Text

There are studies supporting the idea that natural killer (NK) cell alloreactivity in haploidentical hematopoietic stem cell transplantation (haplo-HSCT) decreases in malignant hematological diseases such as acute leukemia, high-risk myelodysplastic syndrome (MDS-AR), and lymphoproliferative syndrome (LPS) [1]. This decrease in alloreactivity can result in several beneficial outcomes in haplo-HSCT, including reduced relapse rates (mediated by the graft vs. tumor (GvT) effect), the decreased incidence of graft vs. host disease (GvHD), and graft rejection (through T cell lysis by donor NK cells), and improved overall survival (OS) rates [1].

## 2. NK Cells

NK cells, along with phagocytes, are part of the innate immune system. They represent the third major lymphoid population in the mononuclear compartment and account for 5–15% of peripheral blood lymphocytes [2]. NK cells play a crucial role in initiating immune responses against viral infections and tumor cells. They share some functions with cytotoxic T lymphocytes (CD8+ T cells), which are involved in adaptive immunity. NK cells are the first lymphoid population to reconstitute after HSCT [3].

Historically, NK cell function was described in 1971 by Cudkowicz and Bennett, initially termed Complement Independent Plaque-Forming Cells. In 1975, their ability to exhibit tumor cytotoxicity was demonstrated in vitro, leading to their classification as NK cells. Subsequently, their antiviral function was discovered in 1978, and in 1987, their potential involvement in mediating graft rejection in bone marrow transplantation was proposed. In 2002, the phenotypes of immature and mature NK cells were defined [4].

**Figure 1:**
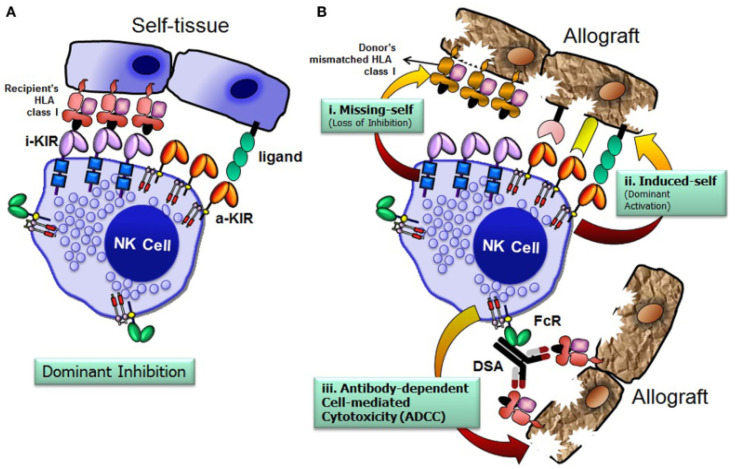
Missing-self theory. (**A**) NK cells avoid attacking healthy cells by detecting self HLA class I through inhibitory receptors. (**B**) However, they can damage allografts if HLA is missing (i), activating signals are strong (ii), or donor-specific antibodies trigger immune responses (iii). FcR, Fc receptor; i-KIR, inhibitory KIR; a-KIR, activating KIR; DSA, donor-specific antibodies. Source: Rajalingam, 2016 [11], under license (CC BY 4.0).

NK cells and cytotoxic T lymphocytes originate from the same lymphoid progenitor and share morphological, phenotypic, and functional characteristics [5]. During their development, NK cells undergo education/licensing, possess receptors, and can expand clonally and generate memory cells during an infection [6]. Unlike T cells and B cells (adaptive immune cells), NK cells recognize healthy cells versus affected cells using different receptor mechanisms. While T cells express activating T cell receptors, NK cells possess both activating and inhibitory receptors. The response of NK cells depends on the stimulus they receive [6,7]. The T cell receptor is not activated if cells express peptides from the individual’s own HLA (Human Leukocyte Antigen) class I system. In contrast, NK cells have inhibitory receptors that recognize HLA class I molecules. Under normal physiological conditions, these inhibitory receptors provide signals that restrain NK cell lytic activity. However, a decrease or alteration in HLA class I molecule expression due to viral infection, tumor transformation, or other forms of stress mitigates the inhibitory influence on NK cells, allowing them to eliminate damaged cells. This phenomenon, described by Ljunggren and Kärre in 1986 as the “missing-self” hypothesis, explains why tumor cells lacking HLA class I molecules are susceptible to destruction by NK cells [4,6,8,9] (Figure 1). This capacity of NK cells to detect missing ligands is the fundamental reason for the induction of GvT effects without promoting GvHD and is supported by in vitro functional studies [10].

## 3. NK Cell Biology

NK cell function fundamentally depends on receptor–ligand interactions. Activating receptors, such as NKG2D, natural cytotoxicity receptors, and DNAM-1, recognize stress-induced ligands like MICA/B and ULBP, or viral glycoproteins, thereby triggering intracellular signaling through Immunoreceptor Tyrosine-based Activation Motif (ITAM)-containing adaptors (DAP10, DAP12, and CD3ζ). The phosphorylation of these ITAMs recruits kinases such as Syk and ZAP70, activating signaling pathways including PLC-γ, PI3K, and MAPK, which in turn induce cytoskeletal reorganization, degranulation, and cytokine release [12,13].

In contrast, inhibitory receptors such as KIRs (killer cell immunoglobulin-like receptors) and the NKG2A/CD94 complex recognize MHC (Major Histocompatibility Complex) class I molecules and recruit phosphatases like SHP-1 and SHIP-1 to suppress activation signals [12,13]. The co-engagement of different receptors, for example, NKG2D together with 2B4, can amplify intracellular signaling, increasing Ca^2+^ flux and cytotoxicity [14].

Regarding cytotoxic mechanisms, NK cells eliminate target cells mainly through three pathways. The first is perforin/granzyme-mediated lysis, in which target recognition induces actin cytoskeleton polarization and the release of secretory lysosomes at the immunological synapse; perforin forms pores in the target cell membrane, allowing granzymes to enter and induce apoptosis via caspase activation [12,15]. The second mechanism involves death receptor pathways, where molecules such as Fas-ligand (FasL), tumor necrosis factor (TNF)-related apoptosis-inducing ligand (TRAIL), and TNF-α interact with their specific receptors on the target cell, activating caspase cascades. In this context, TRAIL is critical for the control of metastasis, while FasL enhances NK activity in the presence of IL-18 [12,16]. Finally, antibody-dependent cellular cytotoxicity occurs when the CD16 (FcγRIII) receptor on NK cells recognizes antibody-opsonized cells, triggering the exocytosis of cytotoxic granules and cytokine production [12,13]. Moreover, the activation of Lymphocyte Function-associated Antigen-1 (LFA-1) on NK cells promotes granule polarization; however, in some instances, it can also inhibit degranulation. This indicates that the interaction between LFA-1 and Intercellular Adhesion Molecules (ICAMs) on target cells may have varying effects on NK cell responses [17]. Additionally, there are studies that indicate that NK cells exhibit spontaneous, time-dependent activation after blood collection, independent of cytokines—an important factor that could significantly influence assessments of resting NK cell activity. Overall, these findings could contribute to the development of novel strategies for activating and expanding the highly cytotoxic CD56^dim^ NK cell subset, offering promising applications in cancer and viral infection therapies [17].

NK cell education and tolerance are maintained through several models. According to the “missing-self” hypothesis, NK cells attack cells lacking MHC class I [8]. The licensing/arming model posits that interaction with self-MHC during development conditions NK cell responsiveness, while the tuning/rheostat model suggests that the intensity of the response is regulated according to the strength of inhibitory receptor signaling, thus balancing activation [18,19]. Notably, NK cell education does not require SHP-1/SHIP-1 activity, supporting the arming model over one based on inhibitory signaling [20].

## 4. Types of NK Cell Receptors

There are several gene families that encode NK cell receptors. Most of these genes are expressed stochastically, meaning that each NK cell clone within an individual may not express the complete set of genes encoding NK receptors present in the genome. Instead, they express a seemingly random combination of these genes [6,11]. As a result, there is an unexpected heterogeneity of NK cell clone subtypes with a variable expression of activating and inhibitory receptors, which explains the rapid detection of target cells [6]. NK receptors can be classified based on their ability to recognize HLA class I molecules. Here, we will focus on the KIR family [6].

## 5. *KIR* Genes

Currently, the *KIR* gene family consists of 15 genes, *KIR2DL1*, *KIR2DL2/L3*, *KIR2DL4*, *KIR2DL5A*, *KIR2DL5B*, *KIR2DS1*, KIR*2DS2*, *KIR2DS3*, *KIR2DS4*, *KIR2DS5*, *KIR3DL1/S1*, *KIR3DL2*, and *KIR3DL3*, and two pseudogenes, *KIR2DP1* and *KIR3DP1*, encoded in the region known as the Leukocyte Receptor Complex (LRC), located on chromosome 19 (19q13.4) [21]. In this region, along with the *KIR* genes, there are other genes that also encode receptors similar to the immunoglobulin family, including two clusters of loci for LILR (Leukocyte Ig-Like Receptor) [22].

The KIR system is exclusive to primates [23]. *KIR* genes are expressed in NK cells and a subset of T lymphocytes [24,25,26]. The expression of *KIR* genes is modulated by multiple factors, including the individual’s KIR haplotype, surrounding class I HLA molecules, and intrinsic genetic factors [3].

As mentioned earlier, during NK cell differentiation, *KIR* expression follows a clonal distribution pattern, allowing the development of different subsets of NK cells in the same individual, displaying different combinations of *KIR* [27].

## 6. Nomenclature and Structure of *KIR* Genes

**Figure 2:**
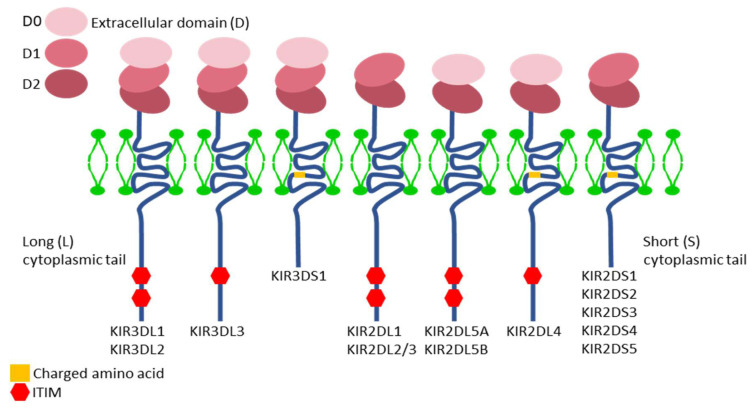
Structure of KIR proteins. Source: Pollock et al., 2022 [30], under license (CC BY-NC-ND 4.0).

The nomenclature of *KIR* genes is based on the structure of the encoded molecules. The first digit following the acronym *KIR* corresponds to the number of extracellular domains (immunoglobulin-like) in the molecule (2D or 3D), and “D” stands for domain. This “D” is followed either by an “L” or an “S,” indicating “Long” or “Short” intracytoplasmic tail, respectively. In cases where the letter “D” is followed by a “P,” it denotes a pseudogene [23,28,29,30] (Figure 2).

When two or more genes have very similar structure and sequence, they are assigned the same number but are differentiated by a letter at the end, as is the case with the genes *KIR2DL5A* and *KIR2DL5B* [31].

## 7. KIR Proteins

KIR proteins or receptors with long intracytoplasmic tails generally contain two domains called ITIM (Immunoreceptor Tyrosine-based Inhibitory Motifs), which have the property of binding to the SH2 domain of signaling molecules, transmitting inhibitory signals in NK cells through dephosphorylation [28] (Figure 2).

KIR proteins with short intracytoplasmic tails provide ITAMs, which require coupling to two DAP12 homodimers to transmit signals and activate the interior of the NK cell through phosphorylation [28].

Thus, KIR receptors with “L” tails transmit inhibitory signals, while KIR receptors with “S” tails trigger NK cell activation. An exception is the *KIR2DL4* gene, which can act as both an activator and inhibitor [6].

## 8. Genetic Organization of *KIR*

*KIR* genes are organized into two haplotypes, A and B, which can exhibit a wide variation in the number and type of *KIR* genes present. Each KIR haplotype, whether A or B, contains four structural genes (framework) (which, with some exceptions, are conserved in all individuals) and define the *KIR* gene cluster. This cluster is flanked by *KIR3DL3* at the centromeric (Cen) level (5’ end) and *KIR3DL2* at the telomeric (Tel) level (3’ end), with *KIR*3*DP1* and *KIR2DL4* positioned centrally in the cluster [11,32]. The Cen and Tel regions are separated by a single sequence known as the recombination site (RS).

**Figure 3:**
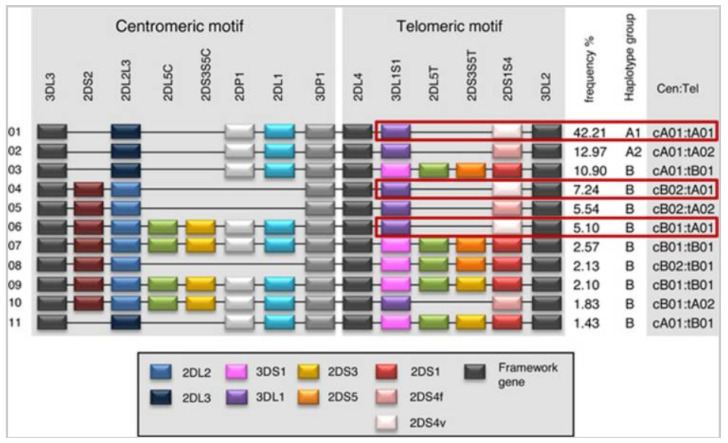
Map of common KIR haplotypes in Caucasian populations. Each rectangle represents a *KIR* gene. Structural genes are shown in black (framework gene, Fw); and pseudogenes (Ps) are in white (2DP1) and gray (3DP1). Source: Traherne et al., 2016 [34], under license (CC BY 4.0).

Group B haplotypes are characterized by the presence of one or more of the following genes: *KIR2DL5A/B*, *KIR2DS1*, *KIR2DS2*, *KIR2DS3*, *KIR2DS5*, and *KIR3DS1.* In contrast, group A haplotypes are characterized by the absence of all these genes. Therefore, group B haplotypes predominantly contain genes encoding activating *KIR*, while group A haplotypes primarily consist of inhibitory genes. In fact, in group A haplotypes, the only possible activation gene is *KIR*2*DS4*, whereas group B haplotypes may have one to five activating *KIR* genes: *KIR2DS1*, *KIR2DS2*, *KIR2DS3*, *KIR2DS5* and *KIR3DS1* [6,33,34] (Figure 3).

It has been observed that NK cells containing group B haplotypes, due to the expression of a higher number of activating *KIR* genes, respond to a greater variety of pathogens [6].

## 9. Polymorphism of *KIR* Genes

In addition to the extensive variation in *KIR* genes across different haplotypes, all *KIR* genes exhibit considerable allelic polymorphism. As of June 2025, a total of 2219 alleles encoding 827 different proteins have been documented in the IPD-*KIR* database [35].

## 10. Combined Inheritance

Due to the proximity between different *KIR* genes, they tend to segregate into haplotypes with a high degree of linkage disequilibrium, suggesting the presence of semi-conserved blocks or associations [13]. The degree of linkage disequilibrium is greater between genes in the Cen region and those in the Tel region than between genes located in different regions (centromere vs. telomere) [6].

*KIR* genes segregate independently from HLA genes, so HLA compatibility does not necessarily imply KIR compatibility [26]. Even in the context of TPH-HLA identical individuals, there is a high probability (approximately 75%) of finding *KIR* gene content mismatch among these HLA-identical individuals [36,37]. In unrelated transplants, only 0.24% of individuals will have matching *KIR* genes [3].

An individual’s genotype can be classified as AA or Bx. The “x” can represent either an A or B haplotype. This is due to the difficulty, in the absence of family studies, of distinguishing whether the other haplotype is A or B in the presence of a B haplotype [38].

## 11. HLA Ligands for KIR Receptors

The ligands with the highest affinity for KIR are class I HLA molecules, particularly the α1/α2 region, making these molecules the most important in recognizing *KIR* genes as ligands, in the following order of importance: HLA-C, HLA-B, and HLA-A [23,39].

**Figure 4:**
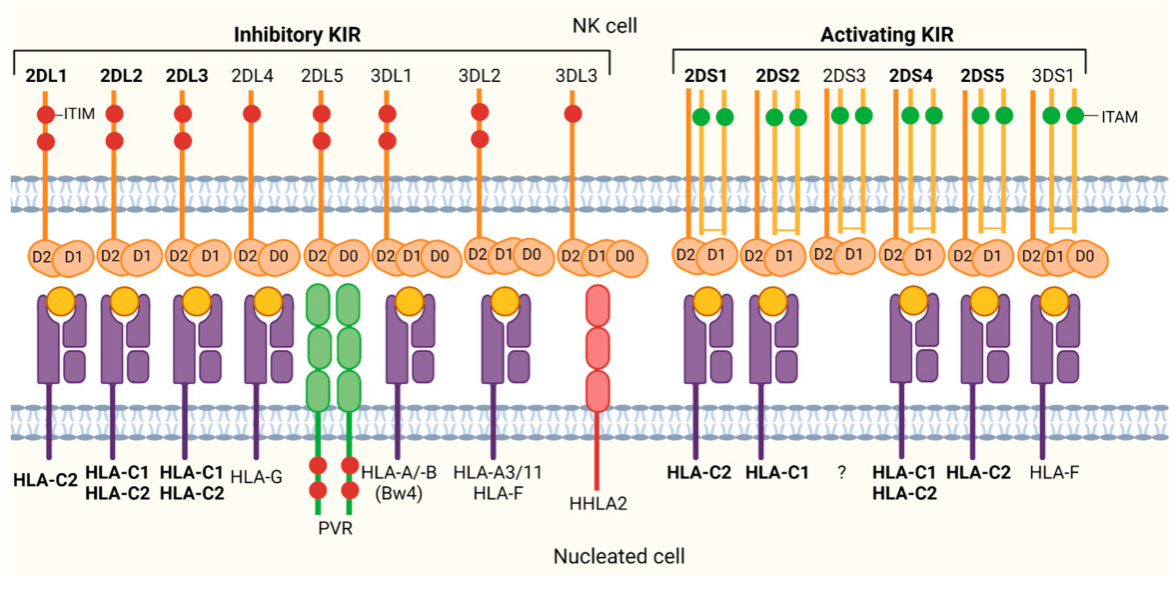
Structure of inhibitory/activating KIRs and their class I HLA ligands. Source: Vollmers et al., 2021 [44], under license (CC BY 4.0).

The specificity of KIR-HLA interaction can be influenced by the presence of certain amino acids at specific positions. Thus, some inhibitory KIR (iKIR) specificities preferentially recognize Lys or Arg amino acids at position 80 of HLA-C ligands [23]. HLA-C molecules have been classified into two groups based on dimorphisms in the α1 domain of the heavy chain. The presence of Ser at position 77 and Asn at position 80 defines group C1, while the presence of Asn at position 77 and Lys at position 80 defines group C2. *KIR2DL1* and *KIR2DS1* molecules interact with HLA-C group C2 allotypes (such as -C*02, -C*04, -C*05, -C*06, -C*12:04, -C*15, -C*16:02, -C*17 and -C*18), while molecules like *KIR2DL2*, *KIR2DL3*, and *KIR2DS2* interact with group C1 allotypes (including -C*01, -C*03, -C*07, -C*12:02, -C*12:03 and C*16:01) [23,40,41,42,43,44] (Figure 4). Position 44 in the first domain of *KIR* is critical in determining its ability to discriminate between the two described HLA-C allotypes [40].

HLA-A and/or HLA-B allotypes that carry a Bw4 motif (determined by the presence of N, D, or S amino acids at residue 77 and IALR, TLLR, or TALR at residues 80–83) are recognized by the *KIR3DL1* receptor, which binds to HLA-Bw4 alleles [45,46,47,48]. Among the five residues in the alpha 1 helix that determine the Bw4 domain, the Ile/Thr dimorphism at residue 80 is the marker that defines the binding affinity for *KIR3DL1*. HLA-A allotypes that are positive for Bw4 and serve as ligands for *KIR3DL1* include A*24:02, *23:01 and *32:01 [43]. The presence of the HLA-Bw4 epitope in an allele of the HLA-B locus leads to greater inhibitory signaling, resulting in better protection against NK cell cytotoxicity compared to when it is present in an HLA-A allele [38].

*KIR3DL2* binds to two HLA-A allotypes, A*03 and A*11, although this receptor is characterized by its low inhibitory capacity, and its interaction with the ligand is highly dependent on the peptide bound to HLA-A [43] (Figure 4).

## 12. Role of NK Cells in HSCT

The graft obtained from the donor in HSCT contains not only hematopoietic stem cells but also immune cells, including mature and immature NK cells. Donor-derived mature NK cells recognize and eliminate tumor cells. They also mediate innate immune responses against viral or bacterial infections and collaborate with T and B lymphocytes in coordinating adaptive immune responses. Additionally, cells derived from the donor’s hematopoietic progenitors can acquire different NK cell phenotypes through receptor maturation and participate in tissue regeneration, such as epithelial tissue [3].

In haplo-HSCT, the high degree of HLA mismatch between the donor and recipient triggers an intense, bidirectional T cell-mediated immune response, wherein donor and/or recipient T cells recognize allogeneic HLA molecules, leading to unacceptably high rates of graft rejection, GvHD, and infections. To overcome this HLA incompatibility barrier inherent to haplo-HSCT, three major transplantation platforms have been developed [49].

### 12.1. Myeloablative Conditioning and T Cell Depletion with Megadoses of CD34+ Cells

T cells are recognized as central mediators of both GvHD and graft failure. Indeed, the T cell content of the graft is directly associated with the risk of GvHD [50,51]. For this reason, strategies were developed to deplete donor T cells prior to allograft infusion. However, subsequent studies revealed that graft failure remained a significant issue, affecting more than 20% of HSCT recipients undergoing T cell depletion. Additionally, the absence of donor T cells reduces the GvH response, increasing the susceptibility of donor grafts to rejection by the host immune system. Graft failure has been associated with the emergence of donor-specific T cells in the recipient that are resistant to conditioning regimens [52].

Beyond immune-mediated rejection, graft failure also appeared to be influenced by the dose of infused hematopoietic progenitors (HPs). In murine models, donor engraftment was achieved by infusing megadoses of CD34+ cells—derived from bone marrow (BM)—in the context of T cell depletion. This approach enhanced the competitive ability of HPs within the BM niche and directly suppressed T cell alloreactivity via CD34+ cells.

In Perugia, Italy, to improve donor engraftment, investigators intensified conditioning protocols using thiotepa, cyclophosphamide (Cy), total body irradiation, and antithymocyte globulin (ATG), along with the infusion of HPs collected from both BM and peripheral blood. This regimen yielded very low rates of GvHD but was associated with poor T cell reconstitution, leading to increased non-relapse mortality (NRM), primarily due to infections—especially viral [17]. Notably, an anti-leukemic effect was preserved despite CD3+ T cell depletion, attributed to the GvT activity of alloreactive NK cells [53].

### 12.2. In Vivo Modulation of T Cell-Replete Grafts Using GIAC Protocol

The GIAC protocol is an acronym derived from the following elements: the G-CSF (granulocyte colony-stimulating factor) mobilization of the donor; intensified immunosuppression using post-transplant cyclosporine A, mycophenolate mofetil, and short-course methotrexate; ATG included in the conditioning regimen to prevent GvHD and facilitate engraftment; and the combination of BM and peripheral blood graft sources [50].

T cells mobilized from BM under G-CSF stimulation exhibit reduced proliferative capacity, decreased Th1 cytokine production, and increased levels of IL-4 (a Th2 cytokine), which, in murine models, has been associated with improved survival and reduced GvHD [54,55].

This platform was first implemented in Beijing, China—at the General Hospital of the Air Force (using BM grafts) and at Peking University (using combined BM and peripheral blood grafts) [56]. The GIAC protocol generally results in consistent primary engraftment with limited relapse rates and favorable disease-free survival. However, it is also associated with higher rates of GvHD, particularly chronic GvHD (cGvHD) [50].

### 12.3. Post-Transplant High-Dose Cyclophosphamide

The concept of immunotolerance induction via Cy dates back to 1959, when Schwartz and Dameshek demonstrated the suppression of antibody formation in rabbits using 6-mercaptopurine [57] in the context of repetitive intravenous human albumin injections as an alloimmunizing stimulus [49].

In the allogeneic setting, Berenbaum and Brown in 1963 were the first to use high-dose Cy to prevent graft rejection in murine skin graft models. They found that administering Cy within four days post-transplant prolonged graft survival, with enhanced efficacy when administered after day two [50,58].

In this approach, unmanipulated BM or peripheral blood grafts are infused, allowing alloreactive T cells to proliferate until day +3 or +4 post-transplant. At that point, high-dose Cy is administered to deplete proliferating T cells in vivo. This early post-transplant Cy (PT-Cy) induces pharmacological immunotolerance by preferentially targeting dividing alloreactive T cells—while sparing non-alloreactive T cells [59].

Several additional mechanisms have been proposed to explain the effects of PT-Cy under specific conditions (i.e., high dose and following BM infusion): [1] the selective elimination of donor and host alloreactive T cells; [2] the induction of regulatory/suppressor T cells; and [3] the intrathymic clonal deletion of donor T cell precursors recognizing recipient antigens, promoting long-term tolerance [60,61].

High-dose Cy administered shortly after haplo-HPs infusion effectively depletes alloreactive T cells from both donor and recipient, thereby preventing both GvHD and graft rejection [62]. However, tolerance cannot be achieved if the recipient has been previously sensitized to donor antigens. Thus, PT-Cy must be administered soon after the initial exposure to alloantigens to exert its immunotolerant effect [49].

The Johns Hopkins group in Baltimore developed a platform for unmanipulated haplo-HSCT using BM-derived HPs and PT-Cy, which has been increasingly adopted since 2008. Recent murine models of HSCT have elucidated novel mechanisms supporting the efficacy of PT-Cy, including the decreased proliferation and impaired survival of CD4+ and CD8+ alloreactive T cells, along with the recovery of regulatory T cells [60].

Cy is an alkylating agent that binds to nitrogenous bases in DNA, causing strand breaks and activating DNA damage sensors that initiate either repair or apoptosis. While Cy acts across all phases of the cell cycle, its effects are most pronounced during the G1 and S phases, thus preferentially targeting proliferating alloreactive T cells. Conversely, HPs exhibit high levels of aldehyde dehydrogenase, rendering them relatively resistant to Cy-induced cytotoxicity and ensuring the safety of hematopoietic engraftment following its administration [63].

Cy is a nitrogen mustard derivative that undergoes hepatic metabolism via cytochrome P450 enzymes to its active form, 4-hydroxycyclophosphamide. This is further hydrolyzed to produce cytotoxic metabolites—phosphoramide mustard and acrolein—which interfere with DNA replication and RNA transcription, leading to cell apoptosis [61].

Beyond the various platforms employed in haplo-HSCT designed to mitigate T cell-mediated alloresponses arising from extensive HLA mismatch between the donor and recipient, multiple algorithmic strategies have been developed to optimize haploidentical donor selection. Among the immunogenetic parameters assessed, donor NK cell alloreactivity has emerged as one of the most extensively investigated criteria, with the goal of selecting donors whose NK cells exhibit potent antitumor activity in the recipient [1].

## 13. Prediction Models of Alloreactivity

There are different models to define the potential alloreactivity of NK cells.

**Figure 5:**
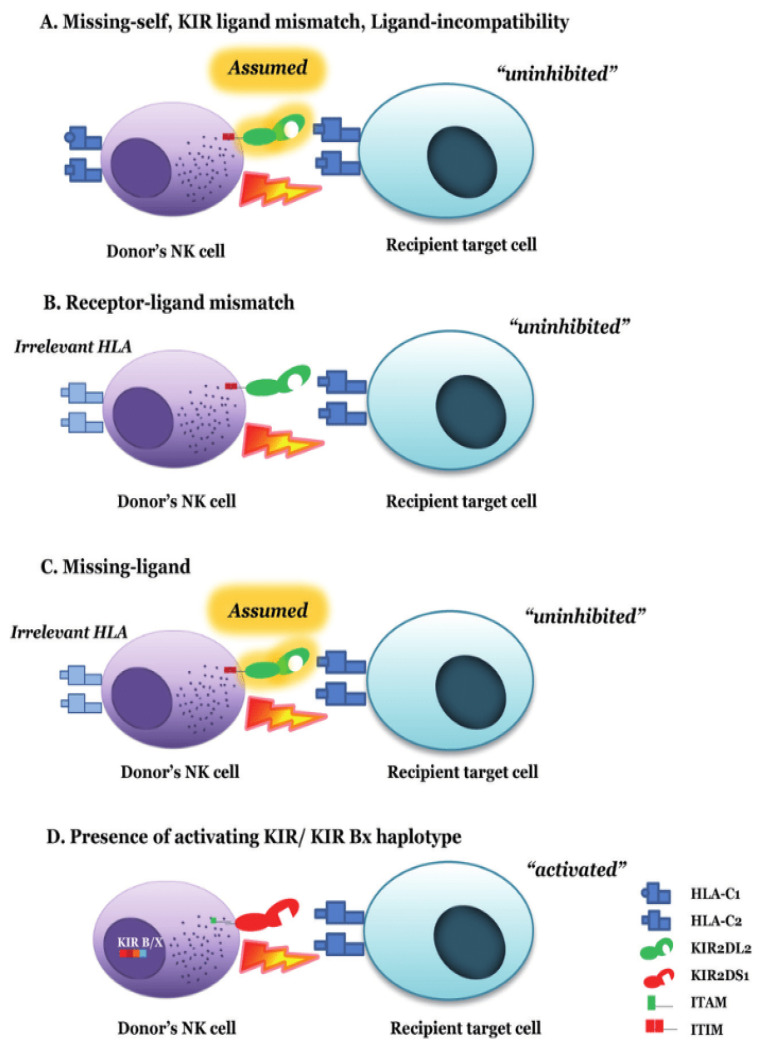
Different alloreactivity NK cells models. Source: Chaisri and Leelayuwat, 2019 [66], under license (CC BY 3.0).

The ligand–ligand incompatibility model (Perugia) is based on the incompatibility of KIR receptor ligands (HLA) between the donor and the patient [53] and derives from the classic “missing-self” hypothesis explained earlier (Figure 5). This model predicts alloreactivity in the direction of GvH if the donor’s NK cells possess ligands that are absent in the recipient’s receptor (C2, C1, Bw4, or A3/A11) [2,64,65,66]. Predicting alloreactivity according to this model requires HLA class I typing (C, B, and A) in both the donor and the patient [53]. This model assumes that if an individual expresses a ligand, they also have the receptor for that ligand [67].

Therefore, a donor will have the potential for NK alloreactivity if they are heterozygous for HLA-C ligands (C1/C2) compared to recipients who are homozygous for these ligands (C1/C1 or C2/C2). Donors who express HLA-Bw4 and/or HLA-A3/A11 epitopes will also potentially be alloreactive if the patient does not have these ligands (i.e., if they are Bw4- and/or A3/A11-negative, respectively). In addition, the donor’s NK cells must be licensed to be functional, meaning that the KIR and its ligand must be expressed in the donor for their NK cells to have effector capacity [26].

The process by which NK cells acquire the necessary competence to recognize foreign entities and eliminate infected or tumor cells is generally referred to as “education” or “licensing.” Although these terms are sometimes used interchangeably, the term “NK cell education” traditionally describes all functional and phenotypic changes induced in NK cells during their development in the BM by the expression of self-HLA class I molecules. The term “licensing” denotes an active mechanism by which mature NK cells acquire a specific response capacity during contact with the interacting cell, depending on the balance/imbalance between the activating signals received and the degree of inhibition caused by the interaction between their KIR receptors and the HLA-I ligands of the target cell [68,69].

Optimal NK cell education following haplo-HSCT occurs when both the donor and recipient coexpress ligands for individual donor iKIRs. Moreover, in patients with acute myeloid leukemia (AML) and MDS, the lowest relapse rates are observed when the donor and host coexpress ligands for all donor iKIRs [70]. Moreover, in the context of haplo-HSCT, the expression of activating receptors has been found to correlate with NK cell education mediated by KIR/MHC class I interactions. Notably, DNAM-1 expression levels were highest in NK cells educated by both donor- and recipient-derived HLA molecules, suggesting distinct regulatory contributions from each. Furthermore, DNAM-1 appears to function synergistically with other activating receptors to promote NK cell functional competence [71]

The receptor–ligand incompatibility model (Memphis) is based on the incompatibility between the donor’s KIR receptor and the patient’s KIR ligand, predicting alloreactivity if the donor has a KIR receptor for which the corresponding ligand is absent in the patient (the “missing ligand” hypothesis) [2]. Predicting alloreactivity according to this model requires the *KIR* genotype of the donor and the HLA class I typing of the patient [2,72] (Figure 5).

The receptor–ligand incompatibility model is based on the observation that not all KIR receptor genes are present in the genome of all individuals, and there can be different expression levels on the surface of NK cells. Unlike the previous model, the presence of KIR is not deduced based on the expressed ligand; instead, it is directly sought.

The absence of the ligand–KIR model in the receptor (missing ligand “assumed”) is defined by the expression of C2 and/or C1, and/or Bw4 in the patient and predicts alloreactivity based on the number of absent KIR ligands in the patient, assuming that the NK cells of most donors will express specific iKIRs for C2, C1, and Bw4 based on their high frequency in the population (Figure 5). This model only requires HLA class I typing (at an intermediate or high resolution) of the patient [73,74]. It is similar to the receptor–ligand model but differs in that it does not consider any HLA/KIR typing of the donor [75].

The *KIR*-*KIR* (gene–gene) incompatibility model (Nantes) [76] is based on the incompatibility between the donor’s KIR receptor and the patient’s KIR receptor, predicting alloreactivity if the donor has a KIR receptor that is absent in the patient (receptor–receptor or gene–gene incompatibility) (Figure 5). An i*KIR* gene–gene incompatibility is defined as an i*KIR* gene present in the donor but absent in their receptor or vice versa (GvH or HvG -host vs. graft incompatibility, respectively) [26].

Based on this “genetic model,” scoring strategies can be developed according to mismatches between donor and recipient KIRs [10]. However, the clinical impact of KIR mismatching varies depending on the transplant platform. In the setting of T-cell-repleted haplo-HSCT with PT-Cy, iKIR mismatching has been associated with improved OS event-free survival and reduced relapse rates, particularly in both lymphoid and myeloid malignancies [26,77]. Conversely, in the context of HLA-identical sibling hematopoietic stem cell transplantation, the findings differ, suggesting a protective effect of donor–recipient *KIR* genotype matching against cGvHD and relapse incidence [78].

The KIR haplotype model, proposed by the Stanford group, is based on the *KIR* genotype of both the donor and the patient. The KIR haplotype model classifies donors and recipients into one of two *KIR* genotypes: AA when the individual is homozygous and both haplotypes belong to the A group, or Bx when their *KIR* genotype is composed of one of the combinations indicated in Table 1 [26,38].

Group A and B KIR haplotypes exhibit distinct Cen and Tel gene content motifs. Since the seminal studies by Cooley et al. in the context of HLA-matched and -mismatched T-cell-replete unrelated donor transplantation—which suggested that Cen-B genes confer protection against relapse and improve survival, at least in AML [1]—multiple investigations have sought to elucidate the differential clinical impact of KIR domain localization (Cen vs. Tel) on transplantation outcomes [1].

In the haplo-HSCT setting, findings have been heterogeneous. Dubreuil et al. (2020) underscored the clinical relevance of selecting donors with a Cen-AA KIR haplotype for haplo-HSCT, proposing that these donors carry inhibitory genes associated with enhanced NK cell education and function, thereby reducing relapse risk in myeloid malignancies without increasing the incidence of GvHD [79]. Conversely, among the donor selection scoring systems for haplo-HSCT proposed by Solomon et al. (2018), the selection of B/x donors carrying Cen B motifs—particularly with the presence of *KIR2DS2/2DL2*—was associated with improved overall and disease-free survival [80]. Other studies, however, have not demonstrated a clinical advantage of B/x donors in AA recipients, regardless of the Cen or Tel location of donor KIR genes [77]. Nonetheless, both studies agree that current findings must be interpreted with caution and validated in larger cohorts of haploidentical donor–recipient pairs.

A recent research review on the role of Cen and Tel KIR haplotypes in disease susceptibility—including transplantation contexts—proposes that the Cen/Tel KIR haplotype framework not only serves as a promising predictor of transplant outcomes but also represents a critical tool for refining donor selection strategies across various transplant platforms [81].

The B-content score, which defines the number of Cen and Tel B genes that define the B haplotype, is used to score the B-content score, which can range from 0 to 4 [1] (Table 2). Based on these results, it is classified as “neutral” when there are no or only one haplotype containing B *KIR* genes, “better” when there are two or more B *KIR* genes as long as both are not in the centromere, and “best” when there are two or more B *KIR* genes, with at least two at the centromere [82] (Table 2). This calculation can be performed using the B-content score tool from the IPD-KIR database [35].

Although this calculator was originally developed based on the studies by Cooley et al. [1]—and thus the classification into three groups initially referred to the relapse protection observed in T-cell-replete unrelated donor HSCT for AML—the subsequent literature has demonstrated its broader applicability as a useful tool for donor selection strategies in the haploidentical transplantation setting.

In a review by Oevermann and Handgretinger (2012) focusing on pediatric transplantation, the influence of activating KIRs in the haploidentical context was emphasized [59]. The authors noted that relapse risk was inversely correlated with the KIR B-content score, as defined by the Cooley model [1,59]. Building on this, a subsequent study recommended that *KIR* genotyping be incorporated into donor selection algorithms for children with acute lymphoblastic leukemia undergoing haplo-HSCT, advocating for the preferential selection of KIR haplotype B donors with a high KIR B-content score whenever feasible [83]. In the study by Bastos-Oreiro, which focused on haplo-HSCT with PT-Cy, no survival benefit was observed in patients who received grafts from donors with higher KIR B-content (B-content score ≥ 3) compared to those who received grafts from donors with a score of 0, 1, or 2. Specifically, there were no significant differences in the cumulative incidence of mortality (HR, 1.2 [0.7–1.9]), relapse (HR, 1.4 [0.93–2.1]), or NRM (HR, 1.15 [0.86–1.53]) [77]. Escudero et al. reported that, in pediatric patients with acute leukemia or chronic myeloid leukemia (CML), OS was significantly worse when the donor had a KIR B-content score ≥ 2 or belonged to the “better” or “best” donor subgroups (*p* = 0.041 and *p* = 0.029, respectively). Moreover, these donors were associated with a significantly lower probability of leukemia-free survival (*p* < 0.001), although the analysis was conducted in the context of T cell-depleted allogeneic HSCT [84]. However, in the study by Cooley et al., 2010 [1], which included 1409 patients with AML or acute lymphoblastic leukemia (ALL) undergoing unmanipulated allogeneic HSCT, donor KIR B haplotype status was not associated with differences in NRM, acute GvHD (aGvHD), or cGvHD. Nevertheless, a significantly reduced relative risk (RR) of relapse was observed among donors classified as “better” (RR, 0.64; *p* = 0.003) and “best” (RR, 0.33; *p* < 0.001), respectively. Furthermore, donors categorized as “best” were associated with improved leukemia-free survival (RR, 0.70; *p* = 0.007). Notably, these findings were specific to patients with AML and were not replicated in those with ALL [1].

More recently, a retrospective study involving patients with hematological malignancies undergoing haplo-HSCT found that those classified in the “better” and “best” donor categories according to the KIR B-content model exhibited significantly poorer relapse-free survival and OS, due to a higher risk of relapse [85]. Notably, this study showed that “neutral” donors were associated with more favorable outcomes in the setting of T-cell-replete haplo-HSCT with PT-Cy, underscoring the potential risk linked to selecting “better” or “best” donors as defined by this scoring system.

Therefore, there are different ways to predict the degree of KIR alloreactivity between the donor and recipient, but it is unknown which model is the best for each type of transplantation. In addition, in the context of unmanipulated haplo-HSCT, without T-cell depletion in the graft, with PT-Cy, there is no unanimous evidence regarding the level at which NK alloreactivity should be considered within the donor selection algorithm. Some centers with extensive experience in this type of platform consider NK alloreactivity as the last aspect to be assessed [86]. This aspect can be very relevant since patients often have several potential haploidentical donors (an average of two to seven potential donors per patient) according to the experience of Johns Hopkins University [87,88], a pioneering center in the use of this type of haplo-HSCT.

## 14. NK Cell Alloreactivity in T-Cell-Replete Haploidentical Transplantation

The role of NK cell alloreactivity in T cell-replete haplo-HSCT remains controversial, with studies reporting inconsistent outcomes [89]. One early hypothesis proposed that the presence of graft-derived T cells may inhibit NK cell activity, thereby limiting their graft vs. leukemia (GvL) potential [89]. In contrast, Solomon et al. found that mismatched KIR ligands and donors with activating KIR haplotypes (such as B/x haplotype with *KIR2DS2*) were associated with reduced relapse rates and improved disease-free survival [80]. Furthermore, donor NK cells expressing *KIR2DS1* have been linked to the better control of GvHD [90], and donors carrying the KIR Bx haplotype were similarly associated with a decreased risk of GvHD. Additionally, the presence of *KIR2DS2* and *KIR2DS1* genes correlated with lower relapse rates [91]. However, some data have also associated the KIR Bx haplotype with the increased incidence of severe aGvHD [92].

Findings from a large study by the European Society for Blood and Marrow Transplantation (EBMT) involving patients with acute leukemia contradicted earlier observations, revealing that KIR/ligand mismatch was linked to a higher relapse risk and inferior OS [93]. Other investigations have pointed out that PT-Cy may diminish NK cell numbers and functionality, potentially reducing their alloreactivity and clinical impact [94]. Moreover, NK cells derived from stem cell grafts are shaped by HLA class I molecules from both the donor and recipient, which complicates predictions about their response to missing HLA ligands on recipient cells [95]. Supporting this, another study demonstrated that PT-Cy significantly reduces the population of alloreactive NK cells in settings with KIR/HLA incompatibility [96].

These conflicting results may reflect differences in graft source, conditioning protocols, *KIR* genotyping techniques, or the specific models of NK alloreactivity used across studies. For example, Zou et al. observed no correlation between clinical outcomes and NK cell alloreactivity models such as the missing ligand or activating KIR/ligand mismatch models. In contrast, a higher Count Functional iKIR (CF-iKIR) score—defined as a cumulative index incorporating multiple iKIRs and their corresponding ligands—was associated with improved survival [97,98]. Previous work also connected CF-iKIR scores with the incidence of viral infections post-transplant across different patient cohorts [99].

## 15. Mismatched KIR Transplantation and GvHD

Although KIR mismatch between donor NK cells and recipient HLA ligands has been associated with enhanced GvL effects, its role in GvHD appears to be limited or neutral in most clinical settings. Several studies have demonstrated that alloreactive NK cells, particularly those mismatched for iKIR ligands, can suppress GvHD by targeting and eliminating antigen-presenting cells (APCs), thereby indirectly reducing T cell activation [53,100]. Notably, NK cell sensitivity to HLA class I polymorphism is largely confined to hematopoietic cells, while many non-hematopoietic tissues—the primary targets in GvHD—lack the expression of the necessary ligands to activate NK cells. This anatomical and molecular limitation reduces the likelihood of NK-mediated tissue injury, even in the context of KIR mismatch. Consistently, clinical studies involving the adoptive transfer of NK cells in both haplo-HSCT and HLA-matched transplantations have reported minimal or no increase in GvHD, especially when grafts are rigorously T cell-depleted [101,102,103]. To further mitigate the risk of GvHD following mismatched transplantation, standard immunosuppressive regimens remain the cornerstone of prophylaxis. These include PT-Cy, calcineurin inhibitors (e.g., tacrolimus or cyclosporine), sirolimus, and mycophenolate mofetil, which can be used in various combinations depending on the transplant platform and patient risk profile. However, caution is warranted with the use of pre-activated NK cells, particularly those capable of producing proinflammatory cytokines such as IFN-γ and TNF-α, which have been implicated in rare cases of GvHD exacerbation [104]. Therefore, optimal management strategies in platforms using NK cells infusion in diverse contexts (i.e., the enhancement of GvL, salvage viral infection therapy) should include donor selection based on favorable KIR-HLA interactions, rigorous T cell depletion, and cytokine monitoring following NK cell infusion to preserve their antitumor efficacy while minimizing the risk of immunopathology [105].

## 16. Clinical and Therapeutic Use of NK Cells and Research

In the clinical and therapeutic context, NK cells have gained prominence in cancer immunotherapy. Strategies such as CAR (Chimeric Antigen Receptor)-NK cells and antibody-based therapies (e.g., anti-CD16) have been shown to enhance tumor recognition and destruction, particularly in tumors overexpressing NKG2D ligands [106,107]. In antiviral defense, NK cells recognize viral proteins such as m157 from MCMV via Ly49H and produce IFN-γ to limit viral replication; however, some viruses, such as CMV, can evade NK cell responses by downregulating stress ligand expression [12,108]. NK cell dysfunction is associated with increased susceptibility to viral infections (such as HPV and HSV) and certain cancers [15].

In the HSCT context, ongoing studies are exploring the use of ex vivo-expanded NK cell products to further improve clinical outcomes. In a phase II trial, Naik et al. evaluated a novel haplo-HSCT approach using CD45RA-depleted, memory T cell-enriched grafts with NK cell addback in pediatric patients with high-risk hematologic malignancies. The strategy, incorporating a submyeloablative, total body irradiation and serotherapy-free conditioning regimen, resulted in robust early immune reconstitution, low relapse rates, and favorable long-term event-free survival, especially in patients in complete remission. Importantly, NK alloreactivity correlated with reduced severe GvHD, underscoring the potential of immune effector cell modulation in optimizing haplo-HSCT outcomes [109]. Moreover, Lee et al. conducted a phase I clinical trial to assess the safety and feasibility of infusing haploidentical, alloreactive NK cells prior to HLA-matched allogeneic stem cell transplantation in patients with high-risk myeloid malignancies. When feasible, NK cells were selected based on KIR/ligand mismatch and administered following chemotherapy but before stem cell infusion. The strategy was well tolerated, did not impair engraftment, and was not associated with increased GvHD. Notably, higher doses of infused NK cells were associated with improved survival, supporting their potential to enhance GvL activity without exacerbating transplant-related toxicity [110].

Current research is exploring new frontiers, such as metabolic reprogramming to optimize nutrient uptake (glucose and amino acids) and improve NK cell persistence in solid tumors [111], the development of memory-like NK cells through preconditioning with IL-12, IL-15, and IL-18 [112], and combination therapies, such as immune checkpoint inhibitors (anti-PD-1) together with NK cell infusions, which show synergistic efficacy [106,107].

Together, all these advances underscore the versatility of NK cells in immune regulation and their growing role in precision medicine and in immunotherapy for transplantation.

## 17. Discussion

Contrasting results have been reported regarding the role of NK cell alloreactivity and KIR/ligand mismatching in haplo-HSCT.

Based on the reviewed data, NK alloreactivity does not appear to be a decisive factor in donor selection for haplo-HSCT using unmanipulated grafts combined with PT-Cy. This finding is consistent with that reported by Ruggeri et al., 2021 [113]. Currently, scientific evidence suggests that high doses of PT-Cy may mitigate or even abrogate the beneficial effects of NK cell alloreactivity previously observed in the T cell-depleted haplo-HSCT model. This notion is partly supported by data demonstrating the loss of the majority of mature NK cells infused during unmanipulated haplo-HSCT following PT-Cy administration, which would presumably diminish the potential alloreactive activity of donor-derived NK cells against the recipient [94]. Consequently, the advantages associated with PT-Cy—such as the simplicity of the procedure and the reduced incidence of moderate-to-severe GvHD [80,87]—may be counterbalanced by the potential loss of NK alloreactivity [94,113]. Nevertheless, in the study by Solomon et al., 2018, which analyzed 208 haplo-HSCT cases treated with PT-Cy, a predictive model of alloreactivity based on receptor–ligand mismatch was associated with improved OS and enhanced leukemia-free survival [80]. Moreover, a large study conducted by the EBMT in patients with acute leukemia challenged earlier findings, showing that KIR/ligand mismatch was actually associated with increased relapse rates and poorer OS [93,98]. One proposed explanation is that PT-Cy, commonly used in this setting, may impair NK cell reconstitution by reducing their numbers and functional capacity, thus diminishing their clinical relevance [94]. Additionally, NK cell education is influenced by the HLA class I environment of both the donor and recipient, complicating the prediction of NK cell responses to missing self-ligands in the recipient [95]. Supporting this, recent evidence has demonstrated that PT-Cy significantly depletes the population of alloreactive NK cells in the context of KIR/HLA incompatibility [96,98]. Furthermore, there is scientific evidence indicating that the use of peripheral blood as the graft source results in a higher number of T cells in the inoculum, which could potentially diminish the impact of NK cell alloreactivity [113,114]. For instance, Zou et al. found no significant correlation between clinical outcomes and conventional NK alloreactivity models such as the missing ligand model or activating KIR/ligand mismatch. Interestingly, they reported that a higher CF-iKIR score—a composite measure accounting for multiple iKIRs and their cognate ligands—was positively associated with better survival outcomes [97]. Notably, this score has also been linked to the incidence of post-transplant viral infections in prior studies across diverse patient populations [43].

Numerous publications have addressed this topic; however, as previously outlined, results regarding the role of NK cell alloreactivity in haplo-HSCT remain contradictory (96,153,155,156,158,208). This inconsistency is likely attributable, at least in part, to the heterogeneity of the underlying hematologic malignancies requiring transplantation, the stem cell source used, donor-intrinsic characteristics, the conditioning regimens applied, *KIR* genotyping strategies, and the particular models used to evaluate NK alloreactivity.

In summary, KIRs play a central role in modulating NK cell alloreactivity in haplo-HSCT, influencing key clinical outcomes such as graft rejection, GvL effects, and GvHD. While their impact can be both beneficial and detrimental, optimizing donor–recipient KIR compatibility and deepening our understanding of the molecular interactions between KIRs and their cognate HLA ligands are essential steps toward improving the efficacy and safety of haplo-HSCT.

## Figures and Tables

**Table 1:** KIR haplotype model with groups assigned based on centromeric and telomeric *KIR* genes. Source: Adapted from Cooley et al., 2010 [1].

Centromeric *(2DS2, 2DL2, 2DL3)*	
Cen-A/A	*2DL3* only
Cen-A/B	*2DL3* with *2DS2* and/or *2DL2*
Cen-B/B	*2DS2* and/or *2DL2;* no *2DL3*
**Telomeric *(3DL1, 3DS1, 2DS1, 2DS4)***	
Tel-A/A	*3DL1* and *2DS4* only
Tel-A/B	*3DL1* and *2DS4* with *3DS1* and/or *2DS1*
Tel-B/B	Lacking *3DL1* and/or *2DS4*

**Table 2:** *KIR-B* gene content score. Donor neutral, better, and best. Source: Adapted from Mehta and Oran, 2019 [82].

*KIR* Genotype	B-Content Score	Cen Haplotypes	Tel Haplotypes	Category
A/A	0	A/A	A/A	*Neutral*
B/x	1	A/A	A/B	*Neutral*
	1	A/B	A/A	*Neutral*
	2	A/A	B/B	*Better*
	2	A/B	A/B	*Better*
	2	B/B	A/A	*Best*
	3	A/B	B/B	*Better*
	3	B/B	A/B	*Best*
	4	B/B	B/B	*Best*

## Data Availability

No new data were created or analyzed in this study.

## References

[1] Cooley S., Weisdorf D.J., Guethlein L.A., Klein J.P., Wang T., Le C.T., Marsh S.G.E., Geraghty D., Spellman S., Haagenson M.D. (2010). Donor selection for natural killer cell receptor genes leads to superior survival after unrelated transplantation for acute myelogenous leukemia. Blood.

[2] Leung W. (2011). Use of NK cell activity in cure by transplant. Br. J. Haematol..

[3] Palmer J.M., Rajasekaran K., Thakar M.S., Malarkannan S. (2013). Clinical relevance of natural killer cells following hematopoietic stem cell transplantation. J. Cancer.

[4] Jiao Y., Huntington N.D., Belz G.T., Seillet C. (2016). Type 1 Innate Lymphoid Cell Biology: Lessons Learnt from Natural Killer Cells. Front. Immunol..

[5] Narni-Mancinelli E., Vivier E., Kerdiles Y.M. (2011). The “T-cell-ness” of NK cells: Unexpected similarities between NK cells and T cells. Int. Immunol..

[6] Rajalingam R. (2011). Human diversity of killer cell immunoglobulin-like receptors and disease. Korean J. Hematol..

[7] Lanier L.L. (2005). NK cell recognition. Annu. Rev. Immunol..

[8] Ljunggren H.G., Kärre K. (1990). In search of the “missing self”: MHC molecules and NK cell recognition. Immunol. Today.

[9] Dębska-Zielkowska J., Moszkowska G., Zieliński M., Zielińska H., Dukat-Mazurek A., Trzonkowski P., Stefańska K. (2021). KIR Receptors as Key Regulators of NK Cells Activity in Health and Disease. Cells.

[10] Dhuyser A., Aarnink A., Pérès M., Jayaraman J., Nemat-Gorgani N., Rubio M.T., Trowsdale J., Traherne J. (2022). KIR in Allogeneic Hematopoietic Stem Cell Transplantation: Need for a Unified Paradigm for Donor Selection. Front. Immunol..

[11] Rajalingam R. (2016). The Impact of HLA Class I-Specific Killer Cell Immunoglobulin-Like Receptors on Antibody-Dependent Natural Killer Cell-Mediated Cytotoxicity and Organ Allograft Rejection. Front. Immunol..

[12] Vivier E., Raulet D.H., Moretta A., Caligiuri M.A., Zitvogel L., Lanier L.L., Yokoyama W.M., Ugolini S. (2011). Innate or adaptive immunity? The example of natural killer cells. Science.

[13] Long E.O., Kim H.S., Liu D., Peterson M.E., Rajagopalan S. (2013). Controlling natural killer cell responses: Integration of signals for activation and inhibition. Annu. Rev. Immunol..

[14] Bryceson Y.T., March M.E., Ljunggren H.-G., Long E.O. (2006). Synergy among receptors on resting NK cells for the activation of natural cytotoxicity and cytokine secretion. Blood.

[15] Orange J.S. (2008). Formation and function of the lytic NK-cell immunological synapse. Nat. Rev. Immunol..

[16] Smyth M.J., Cretney E., Kelly J.M., Westwood J.A., Street S.E.A., Yagita H., Takeda K., van Dommelen S.L.H., Degli-Esposti M.A., Hayakawa Y. (2005). Activation of NK cell cytotoxicity. Mol. Immunol..

[17] Zamai L., Del Zotto G., Buccella F., Gabrielli S., Canonico B., Artico M., Ortolani C., Papa S. (2020). Understanding the Synergy of NKp46 and Co-Activating Signals in Various NK Cell Subpopulations: Paving the Way for More Successful NK-Cell-Based Immunotherapy. Cells.

[18] Orr M.T., Lanier L.L. (2010). Natural killer cell education and tolerance. Cell.

[19] Elliott J.M., Yokoyama W.M. (2011). Unifying concepts of MHC-dependent natural killer cell education. Trends Immunol..

[20] Kim S., Poursine-Laurent J., Truscott S.M., Lybarger L., Song Y.-J., Yang L., French A.R., Sunwoo J.B., Lemieux S., Hansen T.H. (2005). Licensing of natural killer cells by host major histocompatibility complex class I molecules. Nature.

[21] Trowsdale J. (2001). Genetic and functional relationships between MHC and NK receptor genes. Immunity.

[22] Kuroki K., Furukawa A., Maenaka K. (2012). Molecular recognition of paired receptors in the immune system. Front. Microbiol..

[23] Barquera D.T.-G.R. (2008). Receptores de células NK (KIR): Estructura, función y relevancia en la susceptibilidad de enfermedades. Rev. Inst. Nal. Enf. Resp. Mex..

[24] Dupont B., Selvakumar A., Steffens U. (1997). The killer cell inhibitory receptor genomic region on human chromosome 19q13.4. Tissue Antigens.

[25] Selvakumar A., Steffens U., Dupont B. (1996). NK cell receptor gene of the KIR family with two IG domains but highest homology to KIR receptors with three IG domains. Tissue Antigens.

[26] Symons H.J., Leffell M.S., Rossiter N.D., Zahurak M., Jones R.J., Fuchs E.J. (2010). Improved survival with inhibitory killer immunoglobulin receptor (KIR) gene mismatches and KIR haplotype B donors after nonmyeloablative, HLA-haploidentical bone marrow transplantation. Biol. Blood Marrow Transpl..

[27] Muntasell A., López-Botet M. (2016). Natural Killer Cell-Based Immunotherapy in Acute Myeloid Leukemia: Lessons for the Future. Clin. Cancer Res..

[28] Vilches C., Parham P. (2002). KIR: Diverse, rapidly evolving receptors of innate and adaptive immunity. Annu. Rev. Immunol..

[29] Espeli M., Niederer H.A., Traherne J.A., Trowsdale J., Smith K.G. (2010). Genetic variation, Fcγ receptors, KIRs and infection: The evolution of autoimmunity. Curr. Opin. Immunol..

[30] Pollock N.R., Harrison G.F., Norman P.J. (2022). Immunogenomics of Killer Cell Immunoglobulin-Like Receptor (KIR) and HLA Class I: Coevolution and Consequences for Human Health. J. Allergy Clin. Immunol. Pract..

[31] Gómez-Lozano N., Gardiner C.M., Parham P., Vilches C. (2002). Some human KIR haplotypes contain two KIR2DL5 genes: KIR2DL5A and KIR2DL5B. Immunogenetics.

[32] Martin A.M., Freitas E.M., Witt C.S., Christiansen F.T. (2000). The genomic organization and evolution of the natural killer immunoglobulin-like receptor (KIR) gene cluster. Immunogenetics.

[33] Martin A.M., Kulski J.K., Gaudieri S., Witt C.S., Freitas E.M., Trowsdale J., Christiansen F.T. (2004). Comparative genomic analysis, diversity and evolution of two KIR haplotypes A and B. Gene.

[34] Traherne J.A., Jiang W., Valdes A.M., Hollenbach J.A., Jayaraman J., Lane J.A., Johnson C., Trowsdale J., Noble J.A. (2016). KIR haplotypes are associated with late-onset type 1 diabetes in European-American families. Genes Immun..

[35] IPD-KIR Database. https://www.ebi.ac.uk/ipd/kir/.

[36] Shilling H.G., Guethlein L.A., Cheng N.W., Gardiner C.M., Rodriguez R., Tyan D., Parham P. (2002). Allelic polymorphism synergizes with variable gene content to individualize human KIR genotype. J. Immunol..

[37] Shilling H.G., Young N., Guethlein L.A., Cheng N.W., Gardiner C.M., Tyan D., Parham P. (2002). Genetic control of human NK cell repertoire. J. Immunol..

[38] Middleton D., Gonzelez F. (2010). The extensive polymorphism of KIR genes. Immunology.

[39] Rajagopalan S., Long E.O. (2005). Understanding how combinations of HLA and KIR genes influence disease. J. Exp. Med..

[40] Kulkarni S., Martin M.P., Carrington M. (2008). The Yin and Yang of HLA and KIR in human disease. Semin. Immunol..

[41] Mehta R.S., Rezvani K. (2016). Can we make a better match or mismatch with KIR genotyping?. Hematol. Am. Soc. Hematol. Educ. Program..

[42] Sabouri Ghannad M., Hajilooi M., Solgi G. (2014). HLA-KIR Interactions and Immunity to Viral Infections. Res. Mol. Med..

[43] Pende D., Falco M., Vitale M., Cantoni C., Vitale C., Munari E., Bertaina A., Moretta F., Del Zotto G., Pietra G. (2019). Killer Ig-Like Receptors (KIRs): Their Role in NK Cell Modulation and Developments Leading to Their Clinical Exploitation. Front. Immunol..

[44] Vollmers S., Lobermeyer A., Körner C. (2021). The New Kid on the Block: HLA-C, a Key Regulator of Natural Killer Cells in Viral Immunity. Cells.

[45] Van Bergen J., Trowsdale J. (2012). Ligand specificity of Killer cell Immunoglobulin-like Receptors: A brief history of KIR. Front. Immunol..

[46] Velardi A. (2008). Role of KIRs and KIR ligands in hematopoietic transplantation. Curr. Opin. Immunol..

[47] Rettman P., Willem C., Volteau C., Legrand N., Chevallier P., Lodé L., Esbelin J., Cesbron A., Bonneville M., Moreau P. (2017). Impact of Graft-Versus-Graft Natural Killer Cell Alloreactivity on Single Unit Dominance After Double Umbilical Cord Blood Transplantation. Transplantation.

[48] Lutz C.T. (2014). Human leukocyte antigen Bw4 and Bw6 epitopes recognized by antibodies and natural killer cells. Curr. Opin. Organ. Transpl..

[49] Luznik L., O’Donnell P.V., Fuchs E.J. (2012). Post-transplantation cyclophosphamide for tolerance induction in HLA-haploidentical bone marrow transplantation. Semin. Oncol..

[50] Kanakry C.G., Fuchs E.J., Luznik L. (2016). Modern approaches to HLA-haploidentical blood or marrow transplantation. Nat. Rev. Clin. Oncol..

[51] Kernan N.A., Collins N.H., Juliano L., Cartagena T., Dupont B., O’Reilly R.J. (1986). Clonable T lymphocytes in T cell-depleted bone marrow transplants correlate with development of graft-v-host disease. Blood.

[52] Kernan N.A., Flomenberg N., Dupont B., O’Reilly R.J. (1987). Graft rejection in recipients of T-cell-depleted HLA-nonidentical marrow transplants for leukemia. Identification of host-derived antidonor allocytotoxic T lymphocytes. Transplantation.

[53] Ruggeri L., Capanni M., Urbani E., Perruccio K., Shlomchik W.D., Tosti A., Posati S., Rogaia D., Frassoni F., Aversa F. (2002). Effectiveness of donor natural killer cell alloreactivity in mismatched hematopoietic transplants. Science.

[54] Pan L., Delmonte J., Jalonen C.K., Ferrara J.L. (1995). Pretreatment of donor mice with granulocyte colony-stimulating factor polarizes donor T lymphocytes toward type-2 cytokine production and reduces severity of experimental graft-versus-host disease. Blood.

[55] Zeng D., Dejbakhsh-Jones S., Strober S. (1997). Granulocyte colony-stimulating factor reduces the capacity of blood mononuclear cells to induce graft-versus-host disease: Impact on blood progenitor cell transplantation. Blood.

[56] Huang X.-J., Liu D.-H., Liu K.-Y., Xu L.-P., Chen H., Han W., Chen Y.-H., Wang J.-Z., Gao Z.-Y., Zhang Y.-C. (2006). Haploidentical hematopoietic stem cell transplantation without in vitro T-cell depletion for the treatment of hematological malignancies. Bone Marrow Transpl..

[57] Schwartz R., Dameshek W. (1959). Drug-induced immunological tolerance. Nature.

[58] Berenbaum M.C., Brown I.N. (1963). Prolongation of Homograft Survival in Mice with Single Doses of Cyclophosphamide. Nature.

[59] Oevermann L., Handgretinger R. (2012). New strategies for haploidentical transplantation. Pediatr. Res..

[60] Kwon M., Bailén R., Díez-Martín J.L. (2020). Evolution of the role of haploidentical stem cell transplantation: Past, present, and future. Expert. Rev. Hematol..

[61] Al-Homsi A.S., Roy T.S., Cole K., Feng Y., Duffner U. (2015). Post-transplant high-dose cyclophosphamide for the prevention of graft-versus-host disease. Biol. Blood Marrow Transpl..

[62] Kasamon Y.L., Luznik L., Leffell M.S., Kowalski J., Tsai H.-L., Bolaños-Meade J., Morris L.E., Crilley P.A., O’Donnell P.V., Rossiter N. (2010). Nonmyeloablative HLA-haploidentical bone marrow transplantation with high-dose posttransplantation cyclophosphamide: Effect of HLA disparity on outcome. Biol. Blood Marrow Transpl..

[63] Robinson J., Guethlein L.A., Cereb N., Yang S.Y., Norman P.J., Marsh S.G.E., Parham P. (2017). Distinguishing functional polymorphism from random variation in the sequences of >10,000 HLA-A, -B and -C alleles. PLoS Genet..

[64] Farag S.S., Fehniger T.A., Ruggeri L., Velardi A., Caligiuri M.A. (2002). Natural killer cell receptors: New biology and insights into the graft-versus-leukemia effect. Blood.

[65] Barao I., Murphy W.J. (2003). The immunobiology of natural killer cells and bone marrow allograft rejection. Biol. Blood Marrow Transpl..

[66] Chaisri S., Leelayuwat C. (2019). Natural Killer (NK) Cell Alloreactivities against Leukemic Cells: Functions beyond Defense. Cancer Immunotherapy and Biological Cancer Treatments.

[67] Heidenreich S., Kröger N. (2017). Reduction of Relapse after Unrelated Donor Stem Cell Transplantation by KIR-Based Graft Selection. Front. Immunol..

[68] Bessoles S., Grandclément C., Alari-Pahissa E., Gehrig J., Jeevan-Raj B., Held W. (2014). Adaptations of Natural Killer Cells to Self-MHC Class, I. Front. Immunol..

[69] Vivier E., Ugolini S., Blaise D., Chabannon C., Brossay L. (2012). Targeting natural killer cells and natural killer T cells in cancer. Nat. Rev. Immunol..

[70] Zhao X.-Y., Yu X.-X., Xu Z.-L., Cao X.-H., Huo M.-R., Zhao X.-S., Chang Y.-J., Wang Y., Zhang X.-H., Xu L.-P. (2019). Donor and host coexpressing KIR ligands promote NK education after allogeneic hematopoietic stem cell transplantation. Blood Adv..

[71] Shang Q.-N., Yu X.-X., Xu Z.-L., Cao X.-H., Liu X.-F., Zhao X.-S., Chang Y.-J., Wang Y., Zhang X.-H., Xu L.-P. (2022). Functional Competence of NK Cells via the KIR/MHC Class I Interaction Correlates with DNAM-1 Expression. J. Immunol..

[72] Leung W., Iyengar R., Turner V., Lang P., Bader P., Conn P., Niethammer D., Handgretinger R. (2004). Determinants of antileukemia effects of allogeneic NK cells. J. Immunol..

[73] Lotze M.T., Thomson A.W. (2010). Natural Killer Cells: Basic Science and Clinical Application.

[74] Hsu K.C., Keever-Taylor C.A., Wilton A., Pinto C., Heller G., Arkun K., O’Reilly R.J., Horowitz M.M., Dupont B. (2005). Improved outcome in HLA-identical sibling hematopoietic stem-cell transplantation for acute myelogenous leukemia predicted by KIR and HLA genotypes. Blood.

[75] Beksaç M., Dalva K. (2012). Role of killer immunoglobulin-like receptor and ligand matching in donor selection. Bone Marrow Res..

[76] Gagne K., Brizard G., Gueglio B., Milpied N., Herry P., Bonneville F., Chéneau M.L., Schleinitz N., Cesbron A., Folléa G. (2002). Relevance of KIR gene polymorphisms in bone marrow transplantation outcome. Hum. Immunol..

[77] Oreiro M.B. (2017). Influencia de la Alorreactividad KIR en el Trasplante Alogénico de Progenitores Hematopoyéticos Haploidéntico. Ph.D. Thesis.

[78] Sahin U., Dalva K., Gungor F., Ustun C., Beksac M. (2018). Donor-recipient killer immunoglobulin like receptor (KIR) genotype matching has a protective effect on chronic graft versus host disease and relapse incidence following HLA-identical sibling hematopoietic stem cell transplantation. Ann. Hematol..

[79] Dubreuil L., Maniangou B., Chevallier P., Quéméner A., Legrand N., Béné M.C., Willem C., David G., Alizadeh M., Makanga D.R. (2020). Centromeric KIR AA Individuals Harbor Particular KIR Alleles Conferring Beneficial NK Cell Features with Implications in Haplo-Identical Hematopoietic Stem Cell Transplantation. Cancers.

[80] Solomon S.R., Aubrey M.T., Zhang X., Piluso A., Freed B.M., Brown S., Jackson K.C., Morris L.E., Holland H.K., Solh M.M. (2018). Selecting the Best Donor for Haploidentical Transplant: Impact of HLA, Killer Cell Immunoglobulin-Like Receptor Genotyping, and Other Clinical Variables. Biol. Blood Marrow Transpl..

[81] Elagab E.A., Ibrahim A.M., Shediwah A., Alqahtani S.M., TalbAllah S.M. (2025). Role of Centromeric and Telomeric Haplotypes of Killer-Cell Immunoglobulin-Like Receptors (KIRs) in Disease Susceptibility: A Research Review. Cureus.

[82] Mehta R.S., Oran B. (2019). The Optimal Killer Cell Immunoglobulin-Like Receptor Donor-We Can Recognize, but Can We Search?. Biol. Blood Marrow Transpl..

[83] Oevermann L., Michaelis S.U., Mezger M., Lang P., Toporski J., Bertaina A., Zecca M., Moretta L., Locatelli F., Handgretinger R. (2014). KIR B haplotype donors confer a reduced risk for relapse after haploidentical transplantation in children with ALL. Blood.

[84] Escudero A., Martínez-Romera I., Fernández L., Valentín J., González-Vicent M., Vicario J.L., Madero-Jarabo R., Diaz M.Á., Pérez-Martínez A. (2018). Donor KIR Genotype Impacts on Clinical Outcome after T Cell-Depleted HLA Matched Related Allogeneic Transplantation for High-Risk Pediatric Leukemia Patients. Biol. Blood Marrow Transpl..

[85] Byrnes C.P., Hastings A., Lacej I., Palanicawandar R., Olavarria E., Anand A. (2024). A retrospective analysis to evaluate if KIR B haplotype donors associate with a reduced risk of relapse in patients with haematological malignancies following haploidentical transplantation at the Blood and Bone Marrow Transplant Unit at Hammersmith Hospital ICHNHST. HLA.

[86] Ciurea S.O., Champlin R.E. (2013). Donor selection in T cell-replete haploidentical hematopoietic stem cell transplantation: Knowns, unknowns, and controversies. Biol. Blood Marrow Transpl..

[87] Ciurea S.O., Al Malki M.M., Kongtim P., Fuchs E.J., Luznik L., Huang X.-J., Ciceri F., Locatelli F., Aversa F., Castagna L. (2020). The European Society for Blood and Marrow Transplantation (EBMT) consensus recommendations for donor selection in haploidentical hematopoietic cell transplantation. Bone Marrow Transpl..

[88] Fuchs E.J. (2012). Haploidentical transplantation for hematologic malignancies: Where do we stand?. Hematol. Am. Soc. Hematol. Educ. Program..

[89] Zhao X.-Y., Huang X.-J., Liu K.-Y., Xu L.-P., Liu D.-H. (2007). Reconstitution of natural killer cell receptor repertoires after unmanipulated HLA-mismatched/haploidentical blood and marrow transplantation: Analyses of CD94:NKG2A and killer immunoglobulin-like receptor expression and their associations with clinical outcome. Biol. Blood Marrow Transpl..

[90] Sivori S., Carlomagno S., Falco M., Romeo E., Moretta L., Moretta A. (2011). Natural killer cells expressing the KIR2DS1-activating receptor efficiently kill T-cell blasts and dendritic cells: Implications in haploidentical HSCT. Blood.

[91] Gao F., Ye Y., Gao Y., Huang H., Zhao Y. (2020). Influence of KIR and NK Cell Reconstitution in the Outcomes of Hematopoietic Stem Cell Transplantation. Front. Immunol..

[92] Hosokai R., Masuko M., Shibasaki Y., Saitoh A., Furukawa T., Imai C. (2017). Donor Killer Immunoglobulin-Like Receptor Haplotype B/x Induces Severe Acute Graft-versus-Host Disease in the Presence of Human Leukocyte Antigen Mismatch in T Cell-Replete Hematopoietic Cell Transplantation. Biol. Blood Marrow Transpl..

[93] Shimoni A., Labopin M., Lorentino F., Van Lint M.T., Koc Y., Gülbas Z., Tischer J., Bruno B., Blaise D., Pioltelli P. (2019). Killer cell immunoglobulin-like receptor ligand mismatching and outcome after haploidentical transplantation with post-transplant cyclophosphamide. Leukemia.

[94] Russo A., Oliveira G., Berglund S., Greco R., Gambacorta V., Cieri N., Toffalori C., Zito L., Lorentino F., Piemontese S. (2018). NK cell recovery after haploidentical HSCT with posttransplant cyclophosphamide: Dynamics and clinical implications. Blood.

[95] Boudreau J.E., Liu X.-R., Zhao Z., Zhang A., Shultz L.D., Greiner D.L., Dupont B., Hsu K.C. (2016). Cell-Extrinsic MHC Class I Molecule Engagement Augments Human NK Cell Education Programmed by Cell-Intrinsic MHC Class, I. Immunity.

[96] Willem C., Makanga D.R., Guillaume T., Maniangou B., Legrand N., Gagne K., Peterlin P., Garnier A., Béné M.C., Cesbron A. (2019). Impact of KIR/HLA Incompatibilities on NK Cell Reconstitution and Clinical Outcome after T Cell-Replete Haploidentical Hematopoietic Stem Cell Transplantation with Posttransplant Cyclophosphamide. J. Immunol..

[97] Zou J., Kongtim P., Srour S.A., Greenbaum U., Schetelig J., Heidenreich F., Baldauf H., Moore B., Saengboon S., Carmazzi Y. (2022). Donor selection for KIR alloreactivity is associated with superior survival in haploidentical transplant with PTCy. Front. Immunol..

[98] Luis-Hidalgo M., Planelles D., Piñana J.L., Carbonell J., Amat P., Gómez-Seguí I., Guerreiro M., Caballero A., Torío A., Pascual-Cascón M.J. (2025). Frequency and Distribution of KIR Genotypes of Donors-Recipient Pairs in the Haploidentical Haematopoietic Stem Cell Transplantation Setting: Collaborative Study by the Spanish Working Group in Histocompatibility and Transplant Immunology (GETHIT) and the Spanish Haematopoietic Transplantation and Cell Therapy Group (GETH-TC). HLA.

[99] Boelen L., Debebe B., Silveira M., Salam A., Makinde J., Roberts C.H., Wang E.C.Y., Frater J., Gilmour J., Twigger K. (2018). Inhibitory killer cell immunoglobulin-like receptors strengthen CD8+ T cell-mediated control of HIV-1, HCV, and HTLV-1. Sci. Immunol..

[100] Meinhardt K., Kroeger I., Bauer R., Ganss F., Ovsiy I., Rothamer J., Büttner M., Atreya I., Waldner M., Bittrich M. (2015). Identification and characterization of the specific murine NK cell subset supporting graft- *versus* -leukemia- and reducing graft- *versus* -host-effects. OncoImmunology.

[101] Passweg J.R., Tichelli A., Meyer-Monard S., Heim D., Stern M., Kühne T., Favre G., Gratwohl A. (2004). Purified donor NK-lymphocyte infusion to consolidate engraftment after haploidentical stem cell transplantation. Leukemia.

[102] Miller J.S., Soignier Y., Panoskaltsis-Mortari A., McNearney S.A., Yun G.H., Fautsch S.K., McKenna D., Le C., Defor T.E., Burns L.J. (2005). Successful adoptive transfer and in vivo expansion of human haploidentical NK cells in patients with cancer. Blood.

[103] Rubnitz J.E., Inaba H., Ribeiro R.C., Pounds S., Rooney B., Bell T., Pui C.-H., Leung W. (2010). NKAML: A pilot study to determine the safety and feasibility of haploidentical natural killer cell transplantation in childhood acute myeloid leukemia. J. Clin. Oncol..

[104] Shah N.N., Baird K., Delbrook C.P., Fleisher T.A., Kohler M.E., Rampertaap S., Lemberg K., Hurley C.K., Kleiner D.E., Merchant M.S. (2015). Acute GVHD in patients receiving IL-15/4-1BBL activated NK cells following T-cell–depleted stem cell transplantation. Blood.

[105] Simonetta F., Alvarez M., Negrin R.S. (2017). Natural Killer Cells in Graft-versus-Host-Disease after Allogeneic Hematopoietic Cell Transplantation. Front. Immunol..

[106] Liu E., Marin D., Banerjee P., Macapinlac H.A., Thompson P., Basar R., Nassif Kerbauy L., Overman B., Thall P., Kaplan M. (2020). Use of CAR-Transduced Natural Killer Cells in CD19-Positive Lymphoid Tumors. N. Engl. J. Med..

[107] Rezvani K., Rouce R., Liu E., Shpall E. (2017). Engineering Natural Killer Cells for Cancer Immunotherapy. Mol. Ther..

[108] Sun J.C., Lanier L.L. (2011). NK cell development, homeostasis and function: Parallels with CD8^+^ T cells. Nat. Rev. Immunol..

[109] Naik S., Li Y., Talleur A.C., Selukar S., Ashcraft E., Cheng C., Madden R.M., Mamcarz E., Qudeimat A., Sharma A. (2024). Memory T-cell enriched haploidentical transplantation with NK cell addback results in promising long-term outcomes: A phase II trial. J. Hematol. Oncol..

[110] Lee D.A., Denman C.J., Rondon G., Woodworth G., Chen J., Fisher T., Kaur I., Fernandez-Vina M., Cao K., Ciurea S. (2016). Haploidentical Natural Killer Cells Infused before Allogeneic Stem Cell Transplantation for Myeloid Malignancies: A Phase I Trial. Biol. Blood Marrow Transpl..

[111] Cong J., Wang X., Zheng X., Wang D., Fu B., Sun R., Tian Z., Wei H. (2018). Dysfunction of Natural Killer Cells by FBP1-Induced Inhibition of Glycolysis during Lung Cancer Progression. Cell Metab..

[112] Cooper M.A., Elliott J.M., Keyel P.A., Yang L., Carrero J.A., Yokoyama W.M. (2009). Cytokine-induced memory-like natural killer cells. Proc. Natl. Acad. Sci. USA.

[113] Ruggeri L., Vago L., Eikema D.-J., de Wreede L.C., Ciceri F., Diaz M.A., Locatelli F., Jindra P., Milone G., Diez-Martin J.L. (2021). Natural killer cell alloreactivity in HLA-haploidentical hematopoietic transplantation: A study on behalf of the CTIWP of the EBMT. Bone Marrow Transpl..

[114] Bishara A., De Santis D., Witt C.C., Brautbar C., Christiansen F.T., Or R., Nagler A., Slavin S. (2004). The beneficial role of inhibitory KIR genes of HLA class I NK epitopes in haploidentically mismatched stem cell allografts may be masked by residual donor-alloreactive T cells causing GVHD. Tissue Antigens.

